# Effect of the free healthcare policy on socioeconomic inequalities in care seeking for fever in children under five years in Burkina Faso: a population-based surveys analysis

**DOI:** 10.1186/s12939-022-01732-2

**Published:** 2022-09-01

**Authors:** Sekou Samadoulougou, Mariamawit Negatou, Calypse Ngawisiri, Valery Ridde, Fati Kirakoya-Samadoulougou

**Affiliations:** 1 Centre for Research On Planning and Development (CRAD), Laval University, Quebec, G1V 0A6 Canada; 2 Evaluation Platform On Obesity Prevention, Quebec Heart and Lung Institute, Quebec, G1V 4G5 Canada; 3 Centre de Recherche en Epidémiologie, Biostatistiques Et Recherche Clinique, École de Santé Publique, Université Libre de Bruxelles (ULB), Bruxelles, Belgique; 4 Institute for Research On Sustainable Development, CEPED, IRD-Université de Paris, ERL INSERM SAGESUD, Paris, France

**Keywords:** Socioeconomic inequalities, Healthcare utilization, Children under-5, Burkina Faso

## Abstract

**Background:**

In 2016, Burkina Faso implemented a free healthcare policy as an initiative to remove user fees for women and under-5 children to improve access to healthcare. Socioeconomic inequalities create disparities in the use of health services which can be reduced by removing user fees. This study aimed to assess the effect of the free healthcare policy (FHCP) on the reduction of socioeconomic inequalities in the use of health services in Burkina Faso.

**Methods:**

Data were obtained from three nationally representative population based surveys of 2958, 2617, and 1220 under-5 children with febrile illness in 2010, 2014, and 2017–18 respectively. Concentration curves were constructed for the periods before and after policy implementation to assess socioeconomic inequalities in healthcare seeking. In addition, Erreyger’s corrected concentration indices were computed to determine the magnitude of these inequalities.

**Results:**

Prior to the implementation of the FHCP, inequalities in healthcare seeking for febrile illnesses in under-5 children favoured wealthier households [Erreyger’s concentration index = 0.196 (SE = 0.039, *p* = 0.039) and 0.178 (SE = 0.039, *p* < 0.001) in 2010 and 2014, respectively]. These inequalities decreased after policy implementation in 2017–18 [Concentration Index (CI) = 0.091, SE = 0.041; p = 0.026]. Furthermore, existing pro-rich disparities in healthcare seeking between regions before the implementation of the FHCP diminished after its implementation, with five regions having a high CI in 2010 (0.093–0.208), four regions in 2014, and no region in 2017 with such high CI. In 2017–18, pro-rich inequalities were observed in ten regions (CI:0.007–0.091),whereas in three regions (Plateau Central, Centre, and Cascades), the CI was negative indicating that healthcare seeking was in favour of poorest households.

**Conclusion:**

This study demonstrated that socioeconomic inequalities for under-5 children with febrile illness seeking healthcare in Burkina Faso reduced considerably following the implementation of the free healthcare policy. To reinforce the reduction of these disparities, policymakers should maintain the policy and focus on tackling geographical, cultural, and social barriers, especially in regions where healthcare seeking still favours rich households.

**Supplementary Information:**

The online version contains supplementary material available at 10.1186/s12939-022-01732-2.

## Background

Despite the clarion call for African governments to accelerate progress towards reducing child mortality and improving maternal health [1, 2] via the implementation of effective health interventions, child survival remains an urgent concern in Sub-Saharan Africa (SSA) [3, 4]. Indeed, estimates show that although the infant mortality rate plummeted worldwide between 1990 and 2019, from 93 to 38 under-5 deaths per 1000 live births, more than 50% of these deaths occurred in SSA in 2019 [5].

Low-cost interventions have been consistently proven effective in preventing most under-5 deaths in SSA, but predominant inequalities within countries in the region compromise the effects of these interventions in vulnerable populations [4, 6–9]. The “inverse equity hypothesis” has been suggested as a possible explanation for this compromise wherein new health policies or interventions tend to be used more by wealthier populations or in the wealthier/urban areas, resulting in a lag in the poorer populations and consequently, higher death rates [7–9]. Thus, socioeconomic inequalities plunge disadvantaged subpopulations into a continuous cycle of vulnerability and increase their risk of morbidity and mortality [9, 10].

In Burkina Faso, very low healthcare utilisation was observed in the indigent population [11, 12]. In 2010, only 50% of all children sought healthcare services because of the long distances between health centres and the financial precariousness of households [10]. Recent estimates show that public health centres are used more by sick children from rich households (54%) than those from poorer households (22%) [13]. User fee remains the main source of healthcare financing in the country; only 7% of the population has social health protection coverage, and 40% of the total health expenditure was borne by households in 2018 [14].

**Fig. 1:**
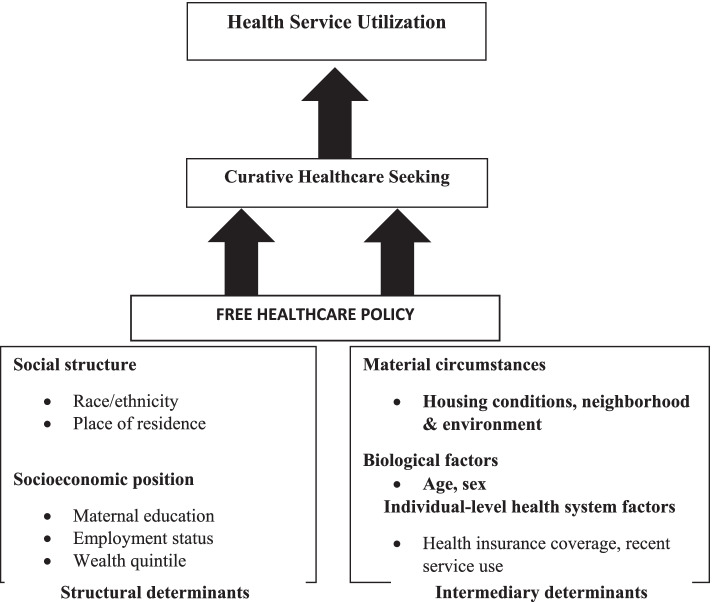
Theoretical Conceptual framework of how the FHCP will impact healthcare seeking and health service utilisation of free healthcare policy. Adapted from WHO (2011) [18]

Increasing healthcare financing reforms, such as free-care policies, have been recommended to improve health outcomes. There is a widespread belief that such reforms will reduce financial hardship from healthcare payments, which, in turn, increases coverage for important child health interventions [15] and contributes to the reduction of infant mortality [16]. It was in this context that in 2016, Burkina Faso adopted the free healthcare policy (FHCP) for women and under-5 children. This policy was intended to cover any consultation for children under 5 years of age, whilst in women, it covered pre-and post-natal consultations, and deliveries. Conceptually, structural determinants and mechanisms that define an individual’s socioeconomic position, together with intermediary determinants, contribute to the occurrence and intensity of ill health. As such, user fee removal via the FHCP will act as an enabling factor, permitting individuals and households to change their health-seeking behaviour as services become more affordable. Healthcare services will thus be used regularly, and consequently, the under-5 mortality rate will reduce [17] (Fig. 1).

In 2019, a study done in Sierra Leone revealed that inequalities were reduced after the implementation of the Free Healthcare Initiative, albeit some inequalities in favour of the wealthy still observed in two districts [3]. Since the implementation of the FHCP in Burkina Faso, the use of child health services has increased in the rural areas [19] and in facilities with higher workforce density [20], and the effect was stronger during the rainy season when need is greater. However, to date, no study has assessed the effect of this policy in reducing socioeconomic inequities among sick children. This study therefore aimed to close this knowledge gap by evaluating the impact of the FHCP on the trend of socioeconomic inequalities in the utilisation of health services for under-5 children seeking care for febrile illness in Burkina Faso.

## Methods

### Setting

Burkina Faso is a West African country with an estimated population of approximately 20 million inhabitants and a surface area of 272,967 km^2^ [21]. Demographic projections show that the population is growing at a rate of 3.1% per year and would reach 28.2 million by 2030(20). This low-income country has an estimated gross domestic product of 786.895 US dollars, and approximately 40.1% of the entire population lives below the poverty threshold [22]. Children under 5 years of age represent 18% of the entire population, and more than 1 out of every 9 children die before the age of 5 years from preventable febrile illnesses such as malaria, pneumonia and diarrhoeal infections [19, 23]. Moreover only 50% of children have access to formal healthcare in Burkina Faso [19].

### Data sources

A secondary data analysis was conducted on three nationally representative surveys from Burkina Faso. A before-after study design was used to compare socioeconomic inequalities among under-5 children with febrile illness seeking care before and after the implementation of the FHCP in Burkina Faso. Data were collected from the Burkina Faso 2010 Demographic and Health Survey (DHS) and the 2014 Malaria Indicator Surveys (MIS) before the implementation of the FHCP, and the 2017–18 MIS after the FHCP implementation. The DHS was only available for 2010; hence, the use of the MIS for 2014 and 2017–18 as the required indicators were available. All surveys used a stratified cluster sampling method for data collection. Overall, 547, 252, and 245 enumeration areas or clusters were selected for the 2010, 2014 and 2017–2018 surveys, respectively. Detailed information regarding the surveys and data collection process has been described previously [21, 24, 25]. This study focused on under-5 children with febrile illness during the two weeks prior to the interview.

### Variables

#### Outcome measure

The outcome variable was care-seeking, which was the proportion of children under five years with fever in the two weeks before the survey, whose caregivers sought treatment in public or private health centres (dichotomous variable, 0 = no and 1 = yes). The healthcare-seeking behaviour was used as a proxy measure of health service utilization (Fig. 1).

#### Independent measures

The independent variables were selected based on the theoretical framework and similar studies [26, 27].

#### Socioeconomic variable

The household wealth index was used as the main socioeconomic variable in this study. The wealth index is a composite indicator of inequalities in household characteristics and was categorized (richest, richer, middle, poor, and poorest) based on the household’s ownership of consumer goods; dwelling characteristics; type of drinking water source; toilet facilities; and other characteristics that relate to a household’s socioeconomic status. The detailed construction and explanation of this variable have been previously described [21, 24, 25].

#### Other variables

The other independent variables constituted mainly sociodemographic variables and determinants of healthcare-seeking for children with fever (biological factors and social structure). Mothers’ ages were re-categorized (15–24 years for younger mothers, 25–34 for middle-aged mothers, and 35–49 for older mothers); likewise, mothers’ education levels into three categories (no education = 0, primary = 1, secondary or more = 2). The age of the child (< 12; 12–35; 36–59 months), head of the household (15–24; 25–34; ≥ 35 years), gender of the household head (male = 0 and female = 1), and religion (Muslim = 1, Christian = 2) were also selected. Other contextual factors were also included, namely, the total number of children (1–2 children = 1, 3–4 children = 2, ≥ 5 children = 3), place of residence (1 = urban, 2 = rural), region (Boucle de Mouhoun, Cascades, Centre, Centre-east, Centre-north, Centre-west, Centre-south, East, Hauts basins, North, Central Plateau, Sahel, South-west), and place of treatment (public, private medical, or other sectors).

### Statistical methods

Statistical analyses were performed using household data with children under five who had experienced an episode of fever two weeks prior to the interview. To summarise the socioeconomic and demographic characteristics of the caregivers and children, descriptive analyses were performed and presented as percentages. To compare the characteristics between the three datasets, the Pearson chi-squared test was used. The analyses were weighted for probability sampling and adjusted for stratification and clustering.

### Measuring socioeconomic inequalities

Socioeconomic inequalities were measured using concentration curves and the concentration index (CI) at the country level (before and after the implementation of the FHCP), which are well-illustrated tools to quantify the socioeconomic inequalities among health-state indicators. At the regional level, CI was used to assess socioeconomic inequalities in the use of healthcare for children under 5.

### Concentration curves

The curves (Lorenz curve) represent the cumulative proportion of ‘treatment-seeking in case of fever for children under 5 years of age’ in relation to the cumulative percentage of ‘wealth’. When computing the cumulative percentage, the wealth quintile variable was ranked from the lowest to the highest. If the dependent variable is evenly distributed, the curve runs diagonally from the bottom left corner to the top right corner (45° line), referred to as the ‘equality line’. In contrast, if the treatment-seeking for fever among children under 5 years is concentrated in the poor, the concentration curve will lie above the line of equality [28].

### Concentration index

This was used to describe the magnitude of inequalities. The index is defined as twice the area between the concentration curve and the equality line (the 45° line) and is represented by the formula below:

$$C=\frac{2}{\mu }cov\left(h,r\right),$$

where $h$ represents healthcare seeking, *μ* represents its mean, $r$ is the fractional rank of an individual in the wealth index distribution, and $cov$ is the covariance between care seeking and the fractional rank of the wealth index [28]. The index varies between -1 and 1, and if the CI is equal to 0, there is no socioeconomic inequality. If the result is positive, the dependent variable is more concentrated among the rich, and the concentration curve will be below the line of equality. In contrast, a negative value means that the dependent variable is more concentrated among the poor, and the concentration curve is above the line of equality [28]. The bounds of the C of a binary health indicator depend on its mean.

As the health indicator varies according to the study period, Erreygers’ normalisation option was selected in STATA. Maps were constructed using QGIS 3.12 software to show the concentration indices for healthcare seeking for under-5 children at the regional level. All analyses were performed using STATA® 14 with a significance level of 0.05.

## Results

In total, 2958 under-5 children with a history of fever were included in 2010, 2617 children in 2014, and 1220 children in 2017–2018, respectively.

### Baseline characteristics of children under 5 years of age and their caregivers

Table 1 illustrates the sociodemographic and household characteristics of the under-5 children and their caregivers. About half (>46%) of the mothers in the three surveys were aged between 25 and 34 years and more than three-quarters lived in the rural areas (>79%). In terms of educational level, the proportion of women with no education was highest in 2010 (81.3%) and lowest in 2017 (75.5%). Moreover, approximately one in every four households (38%) had more than four children aged between 12 and 35 months (Table 1).

**Table 1:** Sociodemographic and household characteristics of study participants

**Characteristics of the population**	**2010**	**2014**	**2017–2018**	
	**n (%)**	**n (%)**	**n (%)**	*p*-value*
**Age of mother (years)**				0.56
15–24	863 (29.5)	749 (28.1)	338 (27.1)	
25–34	1388 (46.5)	1253 (48.7)	598 (48.6)	
≥ 35	707 (24.0)	615 (23.2)	284 (24.3)	
**Total number of children born**				0.047
1–2	960 (32.4)	737 (28.5)	403 (32.9)	
3–4	883 (29.6)	801 (31.1)	379 (31.1)	
≥ 5	1115 (38.0)	1079 (40.4)	438 (36.0)	
**Mother’s education level**				0.005
None	2388(81.3)	2140(79.3)	925(75.5)	
Primary	383 (12.4)	328(14.0)	163(13.6)	
Secondary or higher	186 (6.3)	149 (6.7)	132(10.9)	
**Gender of the household head**				0.21
Male	2759 (93.2)	2468 (94.4)	1158 (95.2)	
Female	199 (6.8)	149 (5.6)	62 (4.8)	
**Wealth index**				0.064
Poorest	482 (16.6)	641(23.2)	278 (24.1)	
Poor	574 (20.0)	602 (21.7)	266 (20.2)	
Middle	700 (23.3)	591 (23.3)	239 (19.2)	
Richer	683 (22.9)	533 (19.1)	245 (19.1)	
Richest	519 (17.3)	250 (14.6)	192 (16.8)	
**Region**				0.011
Boucle de Mouhoun	243 (11.1)	217(10.5)	80 (8.8)	
Cascades	179 (3.8)	194 (3.2)	134 (5.5)	
Centre	165 (8.3)	100 (7.4)	57 (6.1)	
Centre-est	235 (7.9)	220 (8.1)	148 (13.5)	
Centre-nord	170 (6.5)	212 (8.3)	119 (10.1)	
Centre-ouest	275 (8.8)	171 (6.6)	44 (5.2)	
Centre-sud	317 (8.3)	133 (3.0)	45 (2.1)	
Est	159 (5.9)	232 (8.7)	117 (9.8)	
Hauts bassins	294 (14.0)	167 (12.2)	114 (12.9)	
Nord	244 (7.7)	228 (9.3)	121 (9.7)	
Plateau central	237 (5.5)	253 (5.7)	82 (4.2)	
Sahel	143 (5.6)	318 (13.5)	57 (7.6)	
Sud-ouest	297 (6.5)	172 (3.6)	102 (4.4)	
**Place of residence**				0.44
Urban	689 (19.0)	375 (20.2)	178 (15.3)	
Rural	2269 (81.0)	2242 (79.78)	1042 (84.7)	
**Religion**				0.055
Muslim	1770 (62.7)	1747 (69.4)	783 (66.7)	
Christian	1158 (37.3)	868 (30.6)	433 (33.3)	
**Gender of the child**				0.85
Male	1533 (52.0)	1338 (51.4)	626 (51.0)	
Female	1425 (48.0)	1279 (48.6)	594 (49.)	
**Age (months)**				< 0.001
< 12	307 (20.4)	460 (17.0)	275 (22.0)	
12–35	816 (52.8)	1271 (49.1)	541 (44.8)	
36–59	405 (26.8)	869 (33.9)	404 (33.2)	

^* ^Chi-square test for independent proportions

### Socioeconomic inequalities in healthcare seeking

**Fig. 2:**
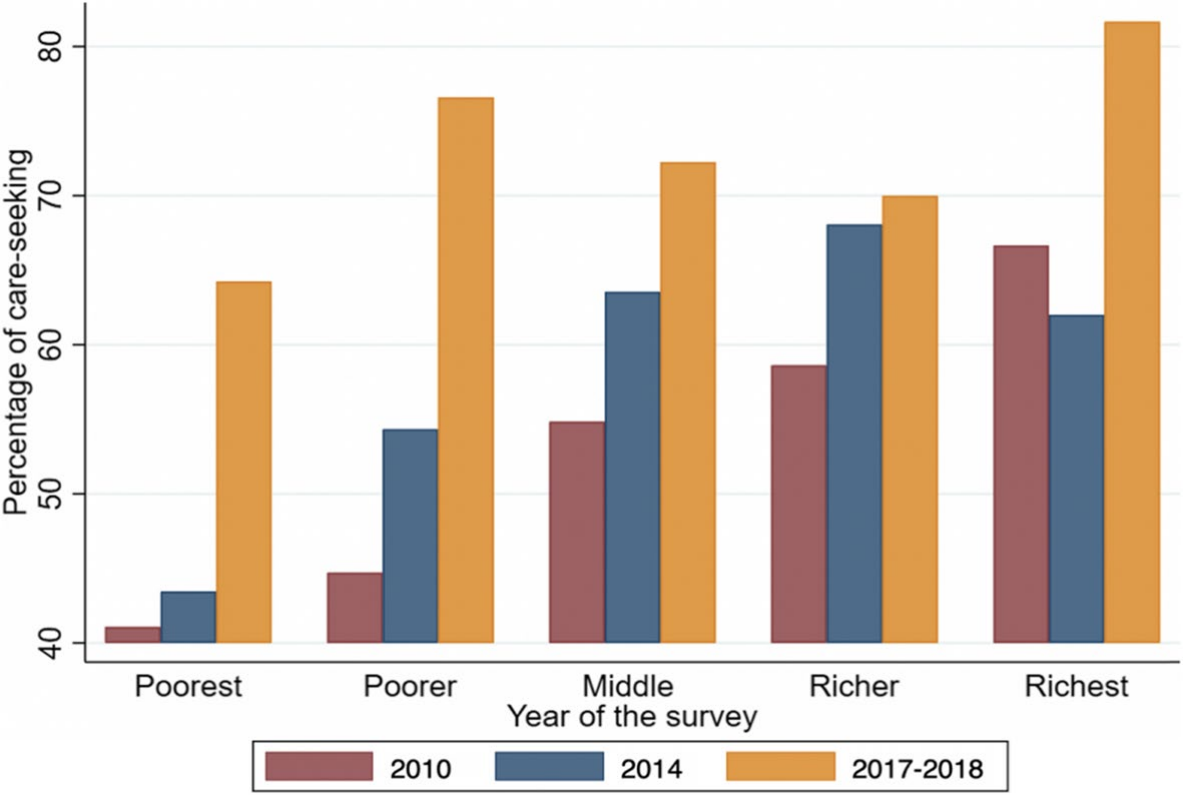
Distribution of health care-seeking for children under five by wealth index and by year

Figure 2 elaborates healthcare seeking for children under five by the wealth index. Except for children from the richest households, healthcare seeking was below 60% in all wealth quintiles in 2010. Overall, there was an increase in healthcare seeking after the implementation of the free healthcare policy. Healthcare seeking for children under 5 years with fever increased from 53.4% (50.4–56.4) in 2010 to 57.5% (54.3–60.7) and 72.3% (68.1–76.2) in 2014 and 2017–2018, respectively.

**Fig. 3:**
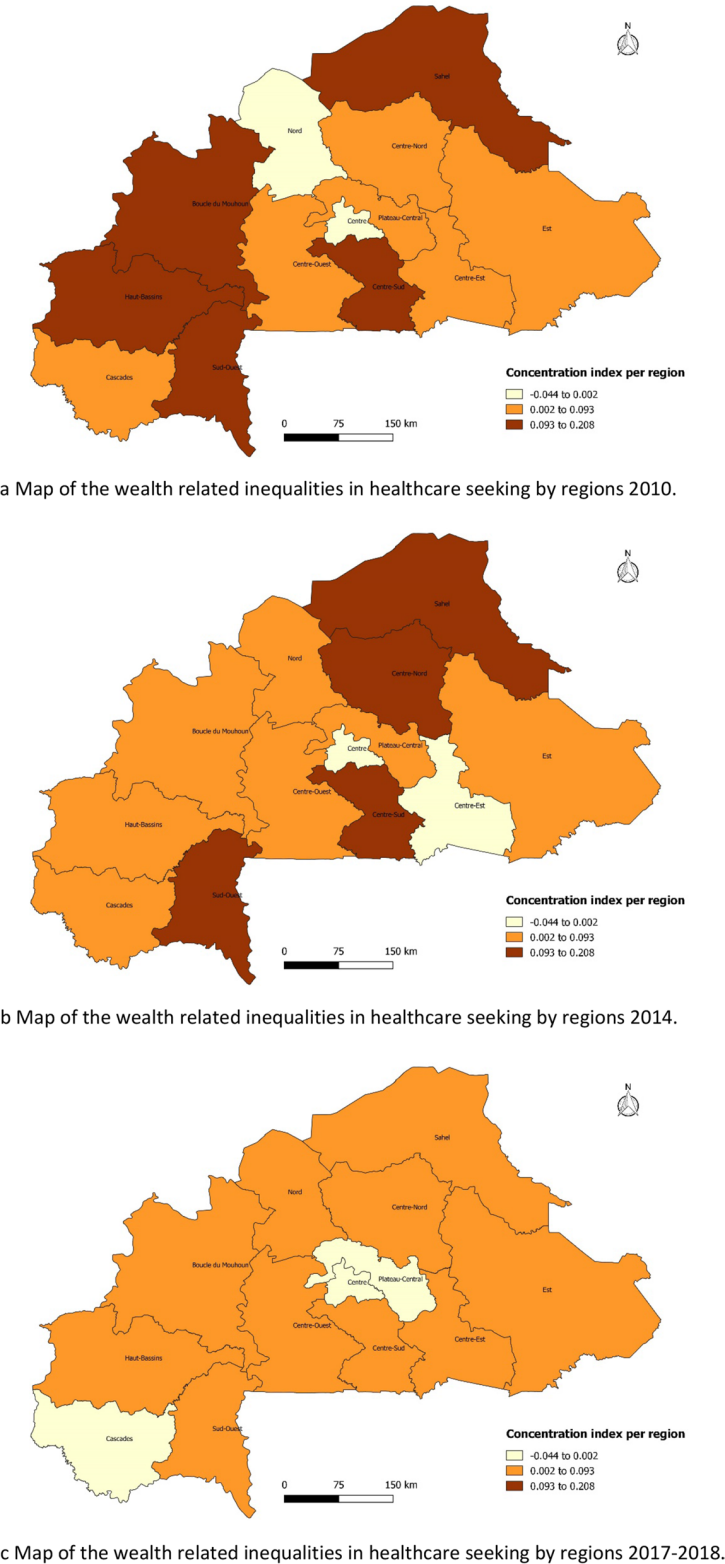
Map of the wealth-related inequalities in healthcare-seeking by region from 2010–2018

Figure 3 represents wealth-related inequalities in healthcare seeking by regions from to 2010–2018. In 2010, care seeking for febrile children favoured wealthy households in all regions except for the Nord and Southwest regions. The CI was high (0.10–0.17) in four regions (Hauts-bassins, Centre, Sahel, and Est), implying that healthcare seeking was in favour of rich households compared to the other regions (Fig. 3a). In 2010, five regions had a high concentration index between 0.093–0.208 which shows that healthcare seeking favoured rich households, whereas in two regions (Nord and Centre), healthcare-seeking favoured poor households (Fig. 3a). In 2014, four regions had a high concentration index between 0.093–0.208 (Fig. 3b) and in 2017–18, no region had a high concentration index between 0.093–0.208. In 10 regions, we observed a pro-rich inequality with a CI between 0.007–0.091, while in three regions (Plateau Central, Centre, and Cascades), the concentration index was negative, showing that healthcare seeking was in favour of poor households (Fig. 3c).

### Concentration index of healthcare seeking for children under 5 with fever

**Fig. 4:**
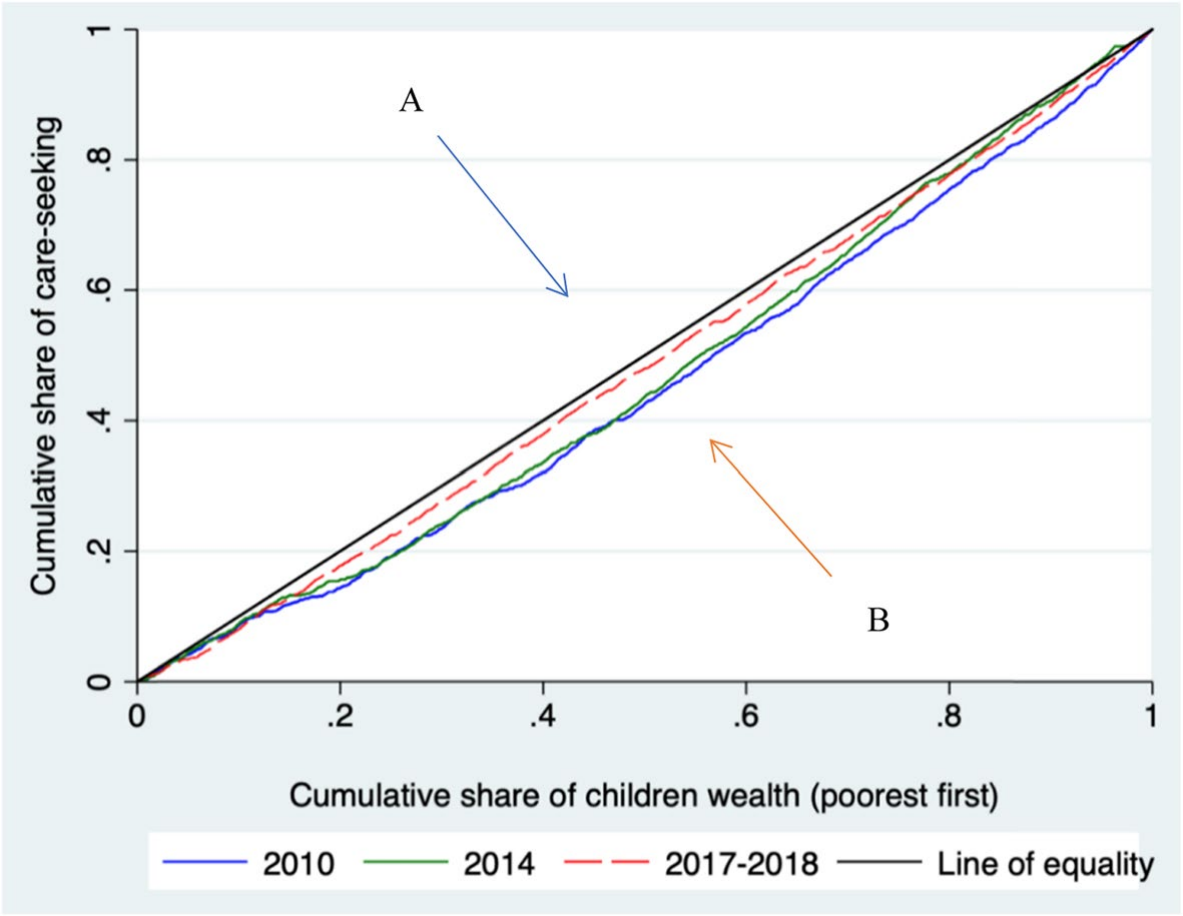
Concentration curves of healthcare-seeking for children under 5 years, 2010–2018. **A** Concentration curve indicating the pro-poor socioeconomic inequality. **B** Concentration curve indicating the pro-rich socioeconomic inequality

Table 2 below demonstrates the concentration index of healthcare-seeking for children under 5 years with fever before (2010–14) and after (2017–18) the implementation of the FHCP. There was a reduction in inequalities in healthcare seeking between rich and poor people. The concentration index value was higher (0.196) in 2010 and decreased progressively in 2014 (0.178) and 2017–18 (0.091) (Fig. 4 and Additional file 1: Annex 1 for region specific analysis).

**Table 2:** The concentration index for healthcare-seeking for children under five by year

**Outcome**	**2010**			**2014**			**2017–18**		
	**CI**	**SE**	***p*-Value**	**CI**	**SE**	***p*-Value**	**CI**	**SE**	***p*-Value**
**Healthcare-seeking**	0.196	0.029	< 0.001	0.178	0.039	< 0.001	0.091	0.041	0.026

*CI* Concentration index, *SE* Standard error

## Discussion

This study examines the impact of the FHCP on socioeconomic inequalities in access to healthcare among under-five children with fever in Burkina Faso and is the first to use nationally representative surveys (DHS and MIS data). Overall, healthcare seeking for under-5 children with fever increased between 2010 and 2017–2018. Before the implementation of the FHCP in 2016, only half of all the under-5 children sought care for fever, and this number increased to almost three-quarters in 2017–2018 after the policy implementation. Furthermore, there was a reduction in inequalities in healthcare seeking for under-5 children between rich and poor people over this same period. However, the inequalities in healthcare-seeking at the country level remained pro-rich after the implementation of the FHCP.

The study results are similar to findings in other SSA countries such as Uganda and Sierra Leone, where user fee abolishment led to an increase in healthcare utilisation, with individuals from low-income households benefiting the most [3, 29]. Inequalities in healthcare seeking between the rich and poor people reduced, was more in favour of poor people and those from the rural areas (more than 79% of the study population) following the implementation of the free healthcare policy. This reduction was marked in the Cascades, Sud-Ouest, and Boucle du Mouhoun regions. This finding could be likely explained by the inverse equity hypothesis wherein the absolute health inequalities at the start of the policy implementation or intervention will increase and with time, decline when the wealthy population is 100% covered before the poor population [7–9].

Our results are consistent with those of studies that report wealth-related inequalities in healthcare access in favour of rich people [26, 27, 29, 30]. Burkina Faso, like most countries in SSA, is characterised by extreme poverty. In addition, most individuals reside in rural, remote areas where the incidence of infectious diseases and maternal and child health problems is very high, coupled with precarious access to healthcare services. Therefore, the care-seeking behaviour in the context of a poorly performing health system is a major problem. In a context where resources are scarce and growing insecurity, providing access to health services for all remains a major challenge in developing countries [31, 32]. In this regard, Burkina Faso has made serious efforts towards ensuring equitable access to child and maternal care services to work towards universal health coverage.

As observed between 2010 and 2014, healthcare affordability (healthcare-seeking) was one of the main challenges in accessing care. This was indicated by the positive concentration index estimated at around 0.196 and 0.178 for 2010 and 2014, respectively, in favour of wealthy individuals. Following the implementation of the FHCP in the second quarter of 2016 for children under five years of age, the inequalities between rich and poor healthcare seeking decreased in 2017–18. This could be explained by, among other things, the reduction of the affordability of care, thus allowing access to healthcare for under-5 children from poor households.

Despite a reduction in inequalities in care-seeking for febrile children under five years of age observed after the implementation of the FHCP, some inequalities like socio-cultural barriers and rural activities in the rainy season remained persistent in poor families,. Therefore, it is important to identify and address the geographical, cultural, and social barriers that affect indigent populations. Policymakers should also utilise “equity-focused strategies” to decrease inequalities. The inverse equity hypothesis can serve as a useful guide, as it has been used for different outcomes to further decrease inequalities. Furthermore, it is noteworthy that national health statistics, as well as several studies conducted in Burkina Faso, have shown that treatment-seeking practices have constantly been increasing in the last few years, even in the absence of interventions [30, 32, 33]. Therefore, user fee removal, the utilisation of community-based health workers for malaria diagnosis and treatment, and the gradual increase in the number of health facilities (from 2278 to 2819 between 2011 and 2019) might accelerate this trend.

The study findings contribute to the ongoing debate about whether the removal of user fees improves care-seeking (or the use of health services) and whether it contributes to a reduction in inequalities between the rich and the poor. To the best of our knowledge, the use of the concentration index is an original method for evaluating the impact of the FHCP on socioeconomic inequalities.

### Limitations

Despite rigorously controlling for several confounders, this study is not without limitations. First, data were obtained from surveys that employed a cross-sectional design which could have resulted in some information bias. Furthermore, factors that may affect care-seeking, such as the readiness of a health facility, severity of illness, and the differences in sociocultural conception of illness and its aetiology, were not considered. Another limitation is the fact that the wealth index is a proxy used in the DHS across many countries whilst the variables used in the Principal Component Analysis (PCA) do not really vary across studies over the years as the nature of household’s assets evolve over time.Therefore, to effectively control and account for all determining factors, further complementary studies combining both qualitative and quantitative approaches are needed to conclude on possible causality.

## Conclusion

This study demonstrated that socioeconomic inequalities in healthcare seeking for under-5 children with febrile illness seeking healthcare in Burkina Faso were considerably reduced following the implementation of the free healthcare policy. To reinforce the reduction of these disparities, policymakers should maintain this policy and tackle the geographical, cultural, and social barriers in regions where healthcare-seeking still favours rich households.

## Supplementary Information

**Additional file 1: Annex 1. **Region specific analysis of** c**oncentration curves of care-seeking for children under 5 years, 2010-2018. **Annex 1a**. Concentration curves of healthcare-seeking for children under 5 years in Centre region, 2010-2018. **Annex 1b**. Concentration curves of healthcare-seeking for children under 5 years in Boucle de Mouhoun, 2010-2018. **Annex 1c.** Concentration curves of healthcare-seeking for children under 5 years in Sud-Ouest region, 2010-2018. **Annex 1d.** Concentration curves of healthcare-seeking for children under 5 years in Cascades region, 2010-2018. **Annex 1e.** Concentration curves of healthcare-seeking for children under 5 years in Centre-Est region, 2010-2018. **Annex 1f.** Concentration curves of healthcare-seeking for children under 5 years in Centre-Nord region, 2010-2018. **Annex 1g.** Concentration curves of healthcare-seeking for children under 5 years in Centre-Ouest region, 2010-2018. **Annex 1h.** Concentration curves of healthcare-seeking for children under 5 years in Centre-Sud region, 2010-2018. **Annex 1i.** Concentration curves of healthcare-seeking for children under 5 years in Est region, 2010-2018. **Annex 1j.** Concentration curves of healthcare-seeking for children under 5 years in Hauts Bassins region, 2010-2018.** Annex 1k.** Concentration curves of healthcare-seeking for children under 5 years in Nord region, 2010-2018. **Annex 1l.** Concentration curves of healthcare-seeking for children under 5 years in Plateau Central region, 2010-2018. **Annex 1m.** Concentration curves of healthcare-seeking for children under 5 years in Sahelregion, 2010-2018.

## Data Availability

The datasets containing individual-level records are freely available from the Demographic and Health Surveys program repository and are accessible after written request: https://dhsprogram.com/data.
