# Supplementary material for: Effect of the free healthcare policy on socioeconomic inequalities in care seeking for fever in children under five years in Burkina Faso: a population-based surveys analysis
Source: Int J Equity Health. 2022 Sep 1;21:124. doi: 10.1186/s12939-022-01732-2 (PMC9438346; doi:10.1186/s12939-022-01732-2)
Supplement: Supplementary file 1 — Additional file 1: Annex 1. Region specific analysis of concentration curves of care-seeking for children under 5 years, 2010-2018. Annex 1a. Concentration curves of healthcare-seeking for children under 5 years in Centre region, 2010-2018. Annex 1b. Concentration curves of healthcare-seeking for children under 5 years in Boucle de Mouhoun, 2010-2018. Annex 1c. Concentration curves of healthcare-seeking for children under 5 years in Sud-Ouest region, 2010-2018. Annex 1d. Concentration curves of healthcare-seeking for children under 5 years in Cascades region, 2010-2018. Annex 1e. Concentration curves of healthcare-seeking for children under 5 years in Centre-Est region, 2010-2018. Annex 1f. Concentration curves of healthcare-seeking for children under 5 years in Centre-Nord region, 2010-2018. Annex 1g. Concentration curves of healthcare-seeking for children under 5 years in Centre-Ouest region, 2010-2018. Annex 1h. Concentration curves of healthcare-seeking for children under 5 years in Centre-Sud region, 2010-2018. Annex 1i. Concentration curves of healthcare-seeking for children under 5 years in Est region, 2010-2018. Annex 1j. Concentration curves of healthcare-seeking for children under 5 years in Hauts Bassins region, 2010-2018. Annex 1k. Concentration curves of healthcare-seeking for children under 5 years in Nord region, 2010-2018. Annex 1l. Concentration curves of healthcare-seeking for children under 5 years in Plateau Central region, 2010-2018. Annex 1m. Concentration curves of healthcare-seeking for children under 5 years in Sahelregion, 2010-2018. [file 12939_2022_1732_MOESM1_ESM.docx]

**Annex 1:** Region specific analysis of **c**oncentration curves of care-seeking for children under 5 years, 2010-2018.

Annex 1a. Concentration curves of healthcare-seeking for children under 5 years in **Centre** region, 2010-2018.


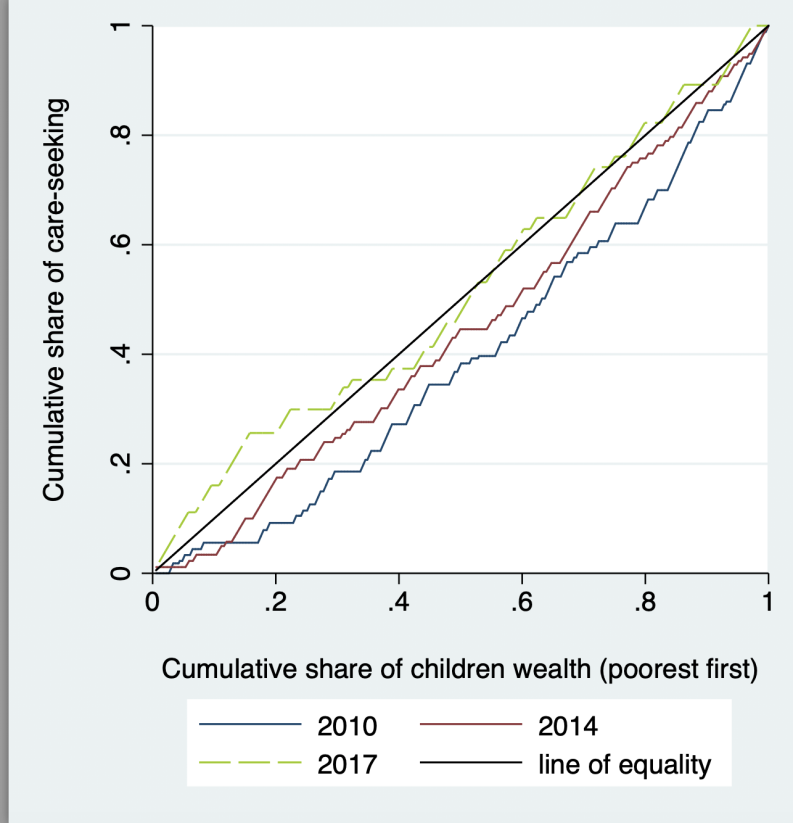


Annex 1b. Concentration curves of healthcare-seeking for children under 5 years in **Boucle de Mouhoun**, 2010-2018.


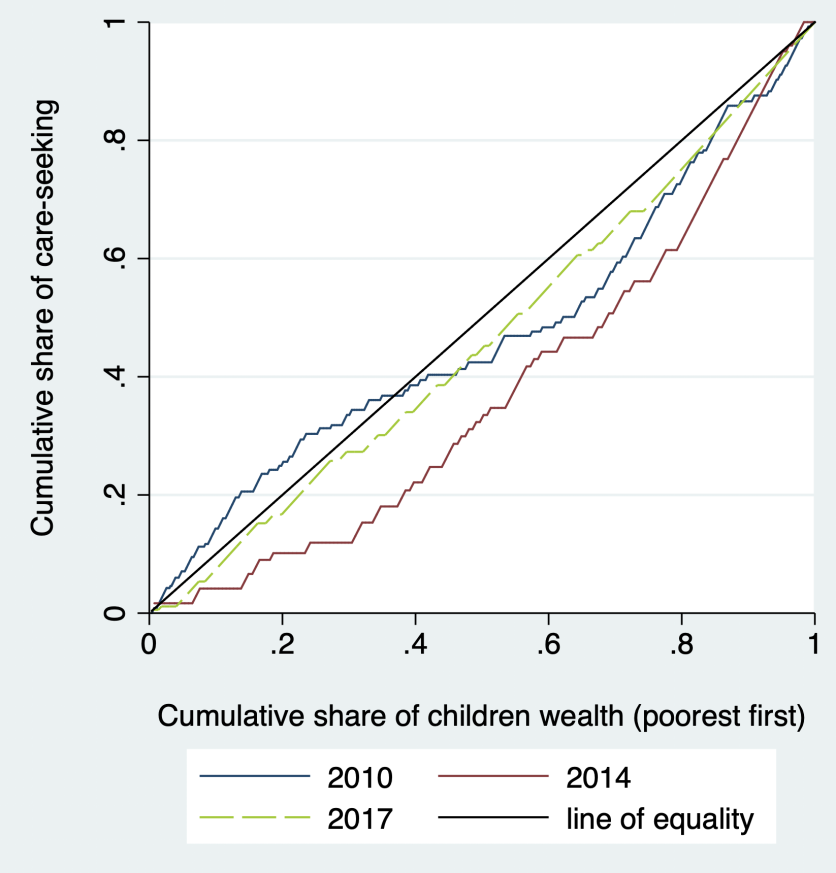


Annex 1c. Concentration curves of healthcare-seeking for children under 5 years in **Sud-Ouest** region, 2010-2018.


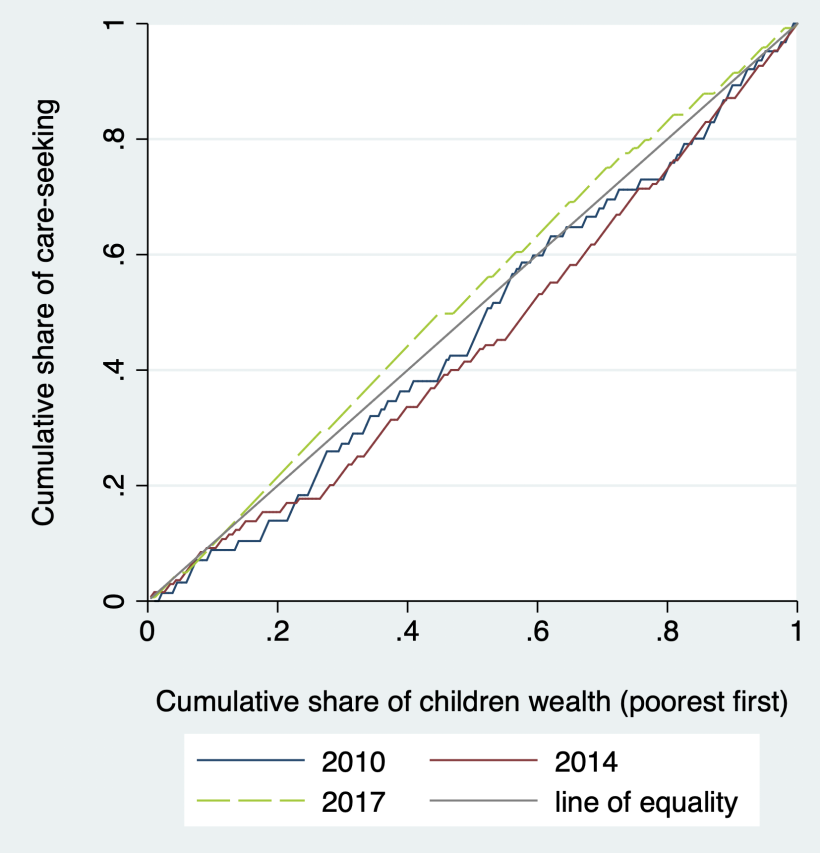


Annex 1d. Concentration curves of healthcare-seeking for children under 5 years in **Cascades** region, 2010-2018.


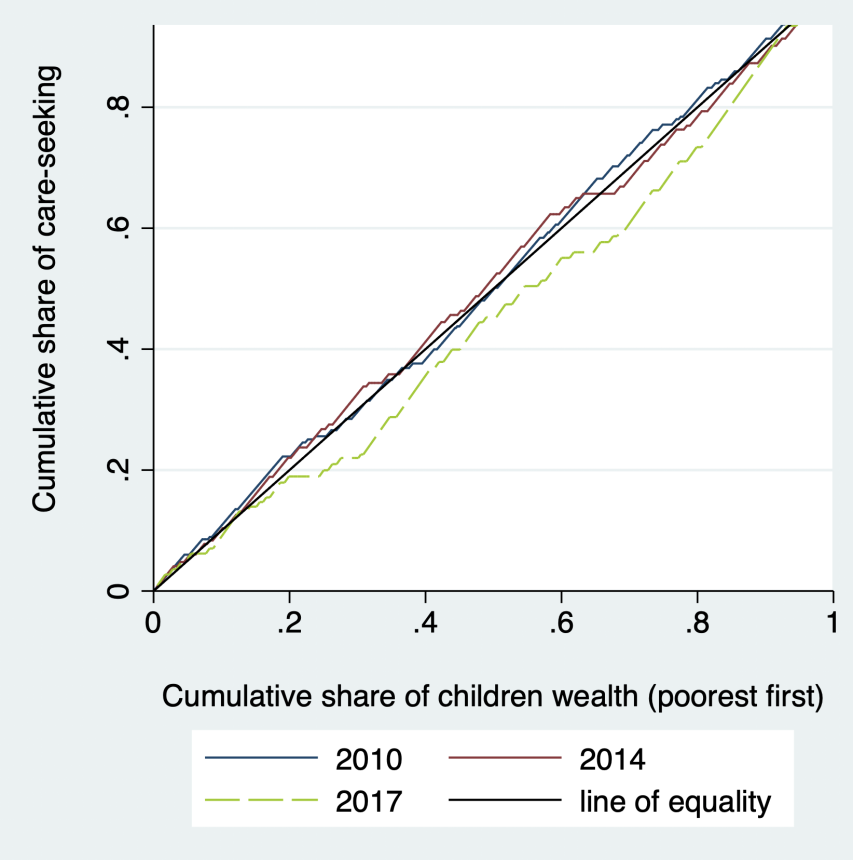


Annex 1e. Concentration curves of healthcare-seeking for children under 5 years in **Centre-Est** region, 2010-2018.


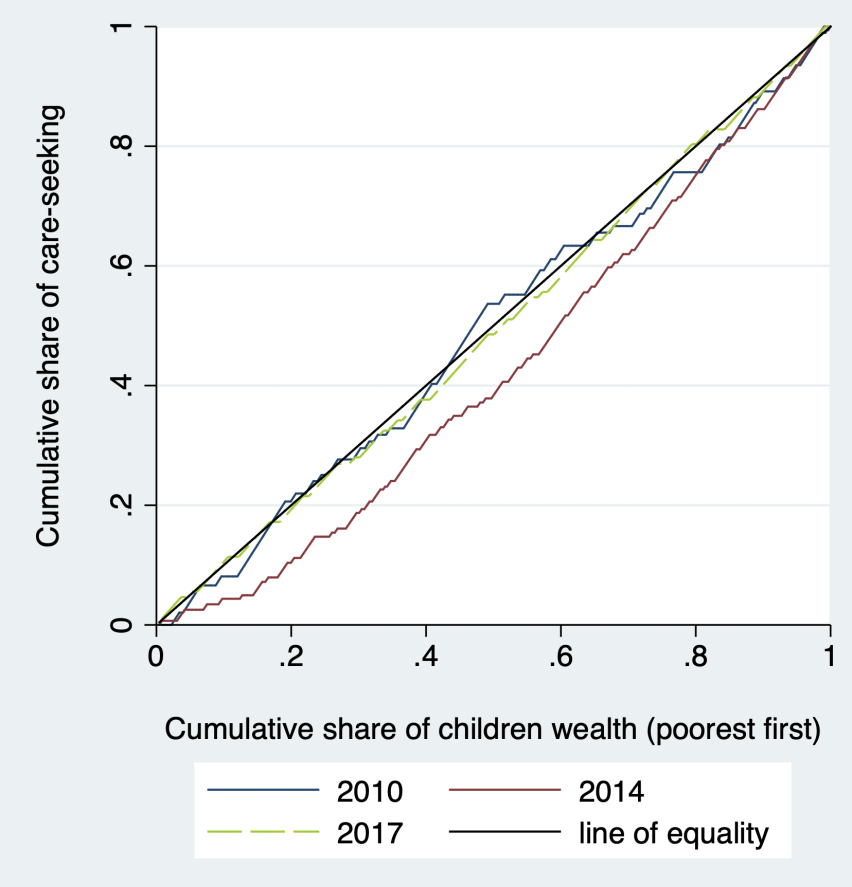


Annex 1f. Concentration curves of healthcare-seeking for children under 5 years in **Centre-Nord** region, 2010-2018.


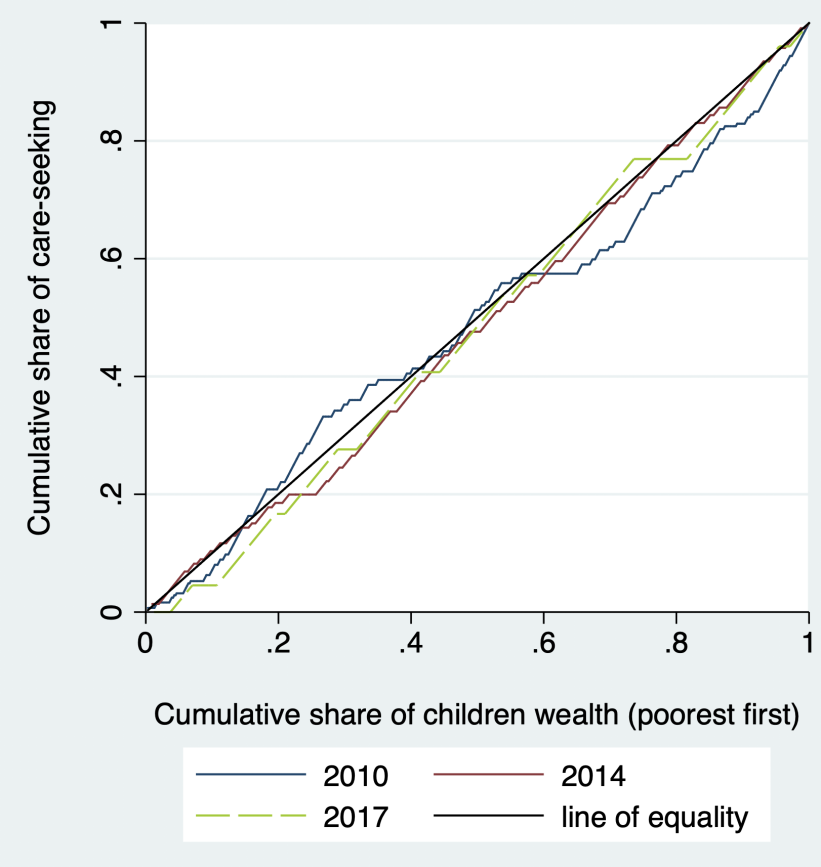


Annex 1g. Concentration curves of healthcare-seeking for children under 5 years in **Centre-Ouest** region, 2010-2018.


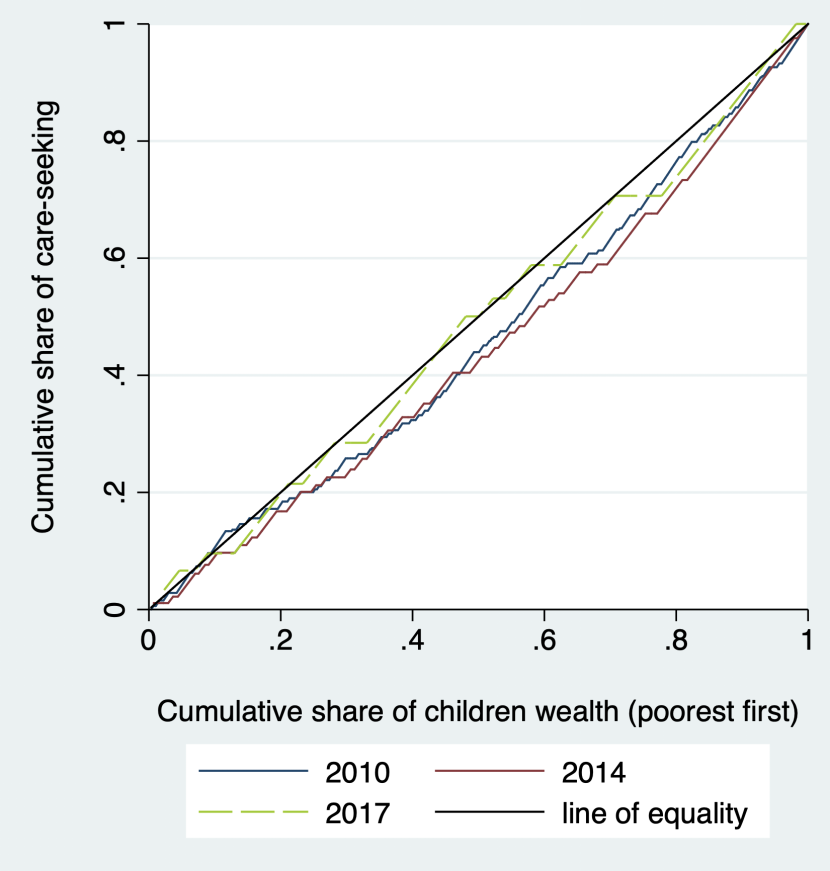


Annex 1h. Concentration curves of healthcare-seeking for children under 5 years in **Centre-Sud** region, 2010-2018.


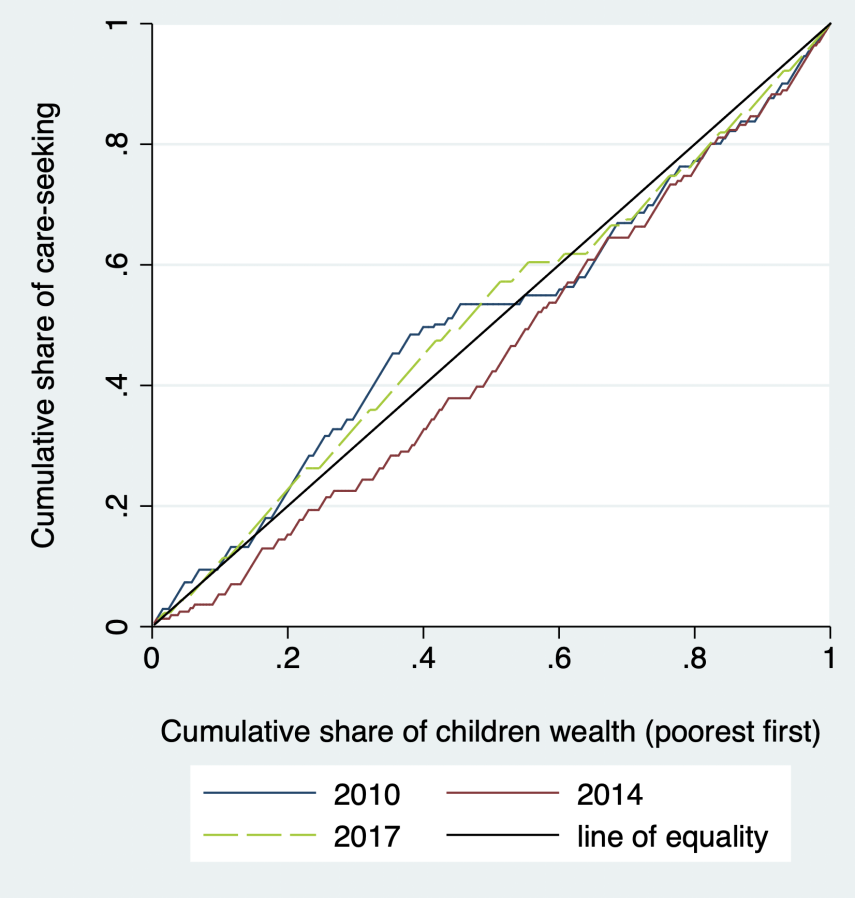


Annex 1i. Concentration curves of healthcare-seeking for children under 5 years in **Est** region, 2010-2018.


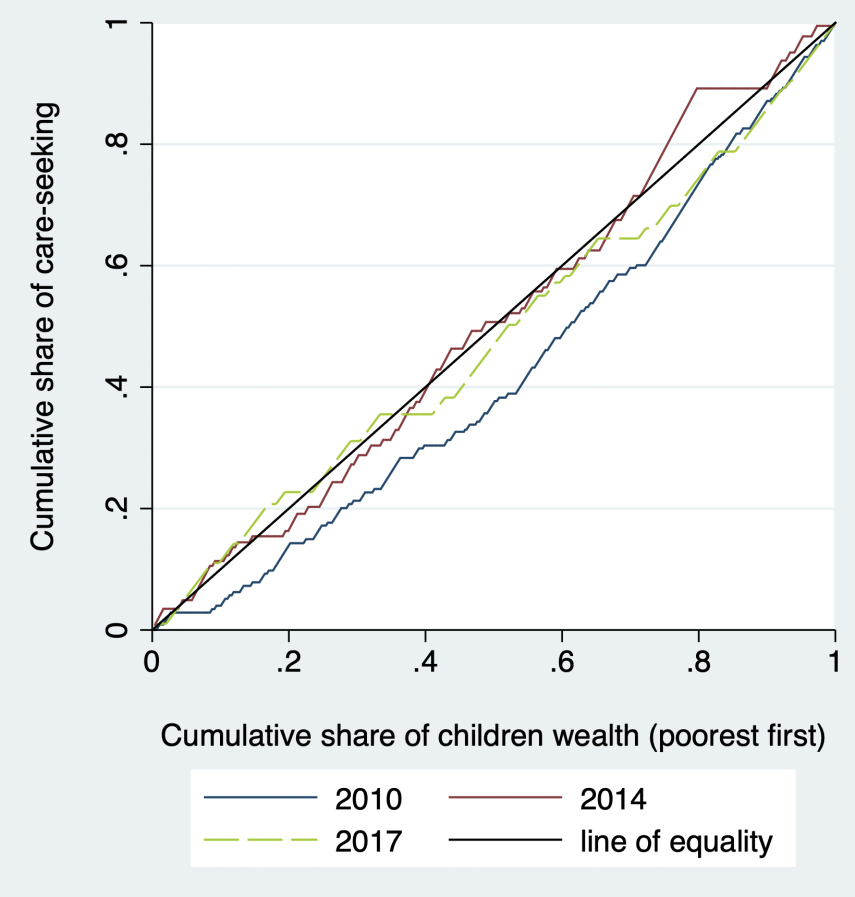


Annex 1j. Concentration curves of healthcare-seeking for children under 5 years in **Hauts Bassins** region, 2010-2018.


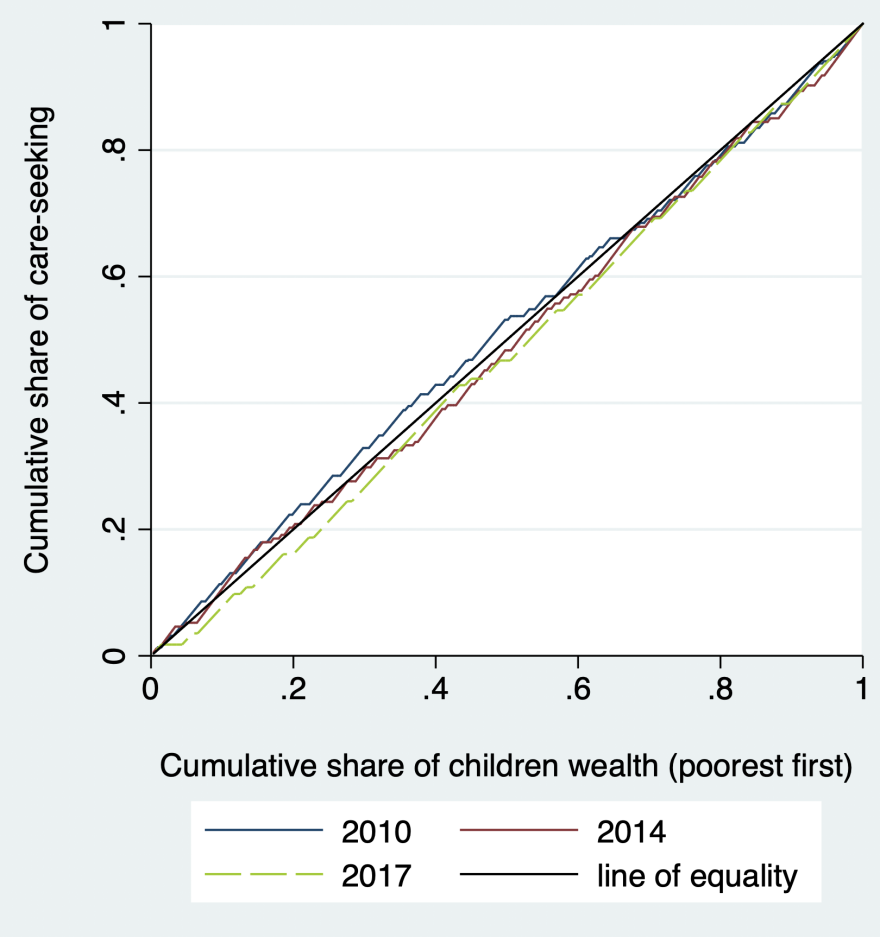


Annex 1k. Concentration curves of healthcare-seeking for children under 5 years in **Nord** region, 2010-2018.


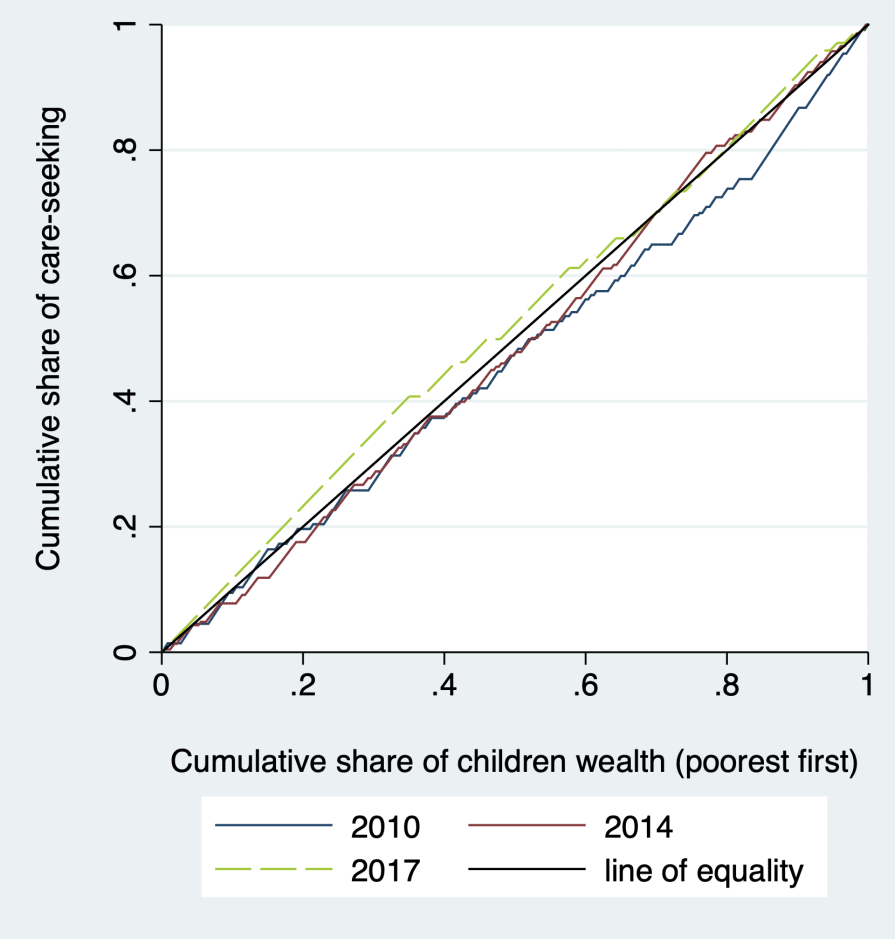


Annex 1l. Concentration curves of healthcare-seeking for children under 5 years in **Plateau Central** region, 2010-2018.


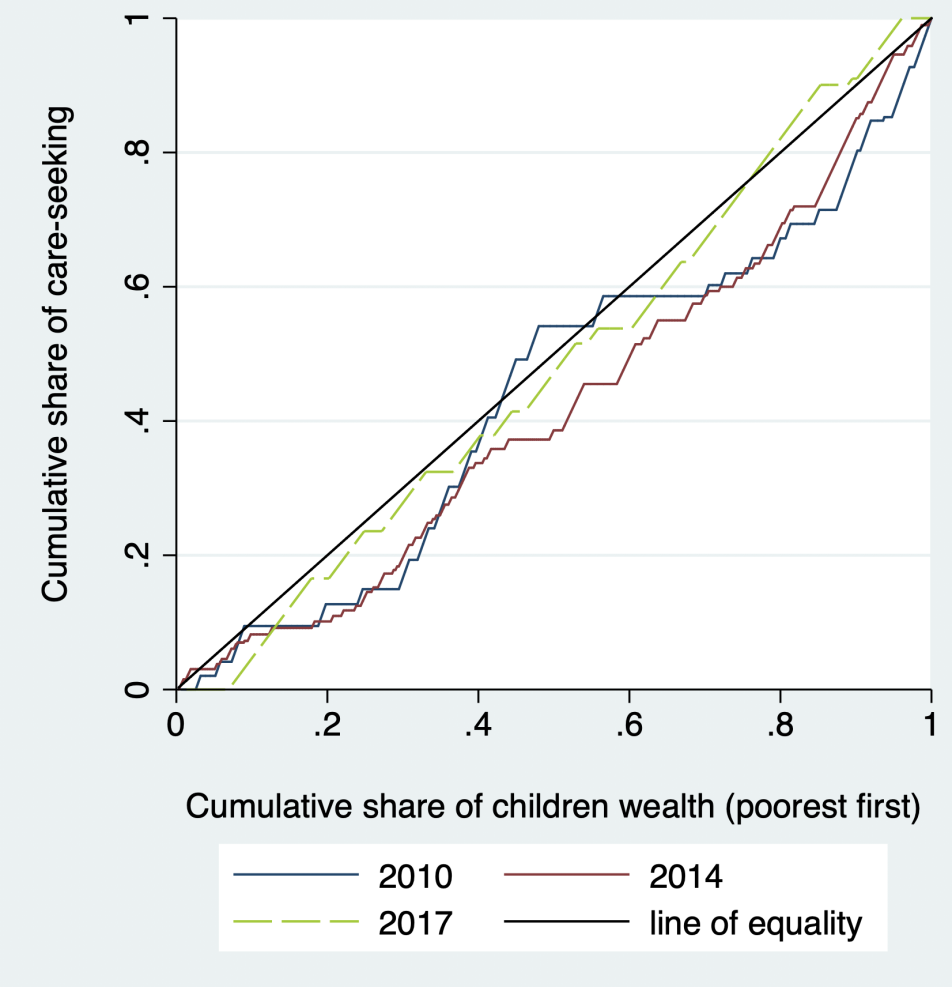


Annex 1m. Concentration curves of healthcare-seeking for children under 5 years in **Sahel** region, 2010-2018.
